# Artificial intelligence-enhanced electrocardiography for prediction of cancer therapy-related cardiac dysfunction

**DOI:** 10.1093/europace/euag191

**Published:** 2026-07-29

**Authors:** Boroumand Zeidaabadi, Claire Glen, Joseph Barker, Oliver H Peck, Yun Y Tan, Ashita Waterston, Iain Squire, Arunashis Sau, Ninian N Lang, Fu Siong Ng

**Affiliations:** National Heart and Lung Institute, Imperial College London, Du Cane Road, London W12 0NN, UK; Glasgow Cardiovascular Research Centre (GCRC), School of Cardiovascular and Metabolic Health, University of Glasgow, Glasgow G12 8TA, UK; National Heart and Lung Institute, Imperial College London, Du Cane Road, London W12 0NN, UK; Imperial College Healthcare NHS Trust, London, UK; Glasgow Cardiovascular Research Centre (GCRC), School of Cardiovascular and Metabolic Health, University of Glasgow, Glasgow G12 8TA, UK; Department of Medical Oncology, Beatson West of Scotland Cancer Centre, NHS Greater Glasgow and Clyde, Glasgow G12 0YN, UK; Department of Medical Oncology, Beatson West of Scotland Cancer Centre, NHS Greater Glasgow and Clyde, Glasgow G12 0YN, UK; Department of Cardiovascular Sciences, University of Leicester and NIHR Leicester Biomedical Research Centre, Leicester LE3 9QP, UK; National Heart and Lung Institute, Imperial College London, Du Cane Road, London W12 0NN, UK; Imperial College Healthcare NHS Trust, London, UK; Glasgow Cardiovascular Research Centre (GCRC), School of Cardiovascular and Metabolic Health, University of Glasgow, Glasgow G12 8TA, UK; National Heart and Lung Institute, Imperial College London, Du Cane Road, London W12 0NN, UK; Imperial College Healthcare NHS Trust, London, UK; Department of Cardiology, Chelsea and Westminster Hospital NHS Foundation Trust, London, UK

**Keywords:** Cardiotoxicity, Artificial Intelligence, Electrocardiography (AI-ECG), Risk stratification, Cancer therapy-related cardiac dysfunction

Targeted therapies inhibiting BRAF and MEK kinases have changed the treatment landscape of *BRAF*-mutated melanoma but are associated with cardiovascular adverse events, particularly cancer therapy-related cardiac dysfunction (CTRCD) and hypertension.^1^ Current guidelines recommend baseline risk stratification using the Heart Failure Association-International Cardio-Oncology Society (HFA-ICOS) risk score to determine surveillance intensity.^2–4^ However, this score relies on traditional clinical variables and static biomarkers, potentially missing subclinical myocardial vulnerabilities. Artificial intelligence-enhanced electrocardiography (AI-ECG) has demonstrated the ability to detect occult structural heart disease and predict future cardiovascular events.^5,6^

We hypothesized that AI-ECG could outperform, or be additive to, the HFA-ICOS score and improve the prediction of CTRCD in patients receiving BRAF/MEK inhibitors (MEKi).

A prospective, longitudinal, cohort study was undertaken among patients with melanoma treated with BRAF and MEKis in a regional cancer network (March 2021 to March 2023). The study design and population have been described previously.^7^ Briefly, patients underwent comprehensive cardiovascular phenotyping, including serial echocardiography and biomarkers at baseline, 4, 12, and 24 weeks. We evaluated two endpoints in line with ICOS definitions: the first occurrence of ‘Any CTRCD’ (defined as Grade ≥ 1) and ‘Moderate/Severe asymptomatic CTRCD or symptomatic CTRCD’ (defined as Grade ≥ 2) at any point during the 6-month follow-up. All patients were assessed prior to commencing BRAF/MEKi therapy; baseline ECGs and HFA-ICOS scores were obtained before treatment initiation.

We used the validated AI-ECG risk estimation (AIRE) platform, trained on >1.2 million ECGs from 189 539 patients at Beth Israel Deaconess Medical Center, Boston, to predict future cardiovascular events.^5^ ECGs were performed at each surveillance visit (baseline, 4, 12, and 24 weeks). For this analysis, we applied the platform's incident heart failure model to the baseline ECGs of the cohort treated with BRAFi or MEKi. We compared the discriminative performance (Harrell’s C-statistic) with 95% confidence intervals (CIs) derived from 1000 bootstrap resamples, area under the curve (AUC), sensitivity, and specificity of three risk stratification strategies:

(1) Clinical (HFA-ICOS score alone); (2) AI-ECG alone; and (3) Combined (HFA-ICOS + AI-ECG). This study was designed and reported in accordance with the EHRA-AI checklist for artificial intelligence in clinical electrophysiology.^8^ The additive prognostic value of AI-ECG was assessed using the partial likelihood ratio test (PLRT); pairwise AUC comparisons were additionally performed using the DeLong test. All analyses were performed in R (version 4.5.2).

The cohort (*n* = 61) had a mean age 59 ± 15 years, and 61% were male. Over a 6-month follow-up, 28 patients (46%) developed ‘Any CTRCD’, primarily mild, asymptomatic strain reductions. Four patients (6.6%) developed ‘Moderate/Severe CTRCD’. All four Grade ≥2 events were first identified at 4 weeks. Two recovered left ventricular ejection fraction (LVEF) to >50% and restarted treatment; one had therapy discontinued for non-cardiovascular reasons (LVEF 53% at 24 weeks); and one developed severe CTRCD (LVEF nadir 37%, partial recovery to 49% at 24 weeks on quadruple therapy). No patient developed symptoms of heart failure.

For the broad endpoint of ‘Any CTRCD’ (Grade ≥1), discrimination was modest across all modalities. The HFA-ICOS score achieved a C-statistic of 0.587 (95% CI: 0.48–0.70), which was not significantly improved by the AI-ECG model (0.596) or the Combined model (0.611). This may reflect the transient and heterogeneous nature of Grade 1 toxicity, which often resolves without intervention and may represent haemodynamic fluctuation rather than structural injury.

In contrast, for the endpoint of ‘Moderate/Severe CTRCD’ (Grade ≥2), the AI-ECG model demonstrated numerically higher predictive value. The C-statistic improved from 0.814 (95% CI 0.585–1.000) with HFA-ICOS alone to 0.890 (0.751–1.000) with AI-ECG alone and 0.905 (0.783–0.963) with the Combined model (PLRT, *P* = 0.030).

At the Youden-optimal threshold the AI-ECG achieved 100% sensitivity/73.7% specificity; AUC 0.906 (0.78–1.00), vs. HFA-ICOS alone (75%/86%; AUC 0.816, 0.58–1.00) and the Combined model (100%/82.5%; AUC 0.917, 0.83–1.00). Adding AI-ECG to HFA-ICOS improved predictive model fit for Moderate/Severe CTRCD; however, the DeLong test for AUC difference was non-significant (Combined vs. HFA-ICOS: *P* = 0.27; AI-ECG vs. HFA-ICOS: *P* = 0.54), indicating an overall trend towards improved discrimination.

Gradient-based saliency maps were generated using the tf-keras-vis^9^ library. Lead-level attribution was quantified by dividing each 310 × 868 pixel image into a 3 × 4 grid and computing the proportion of normalized saliency within each region. Leads I, II, and aVR were identified as the primary leads of model focus (***Figure 1***).

**Figure 1:**
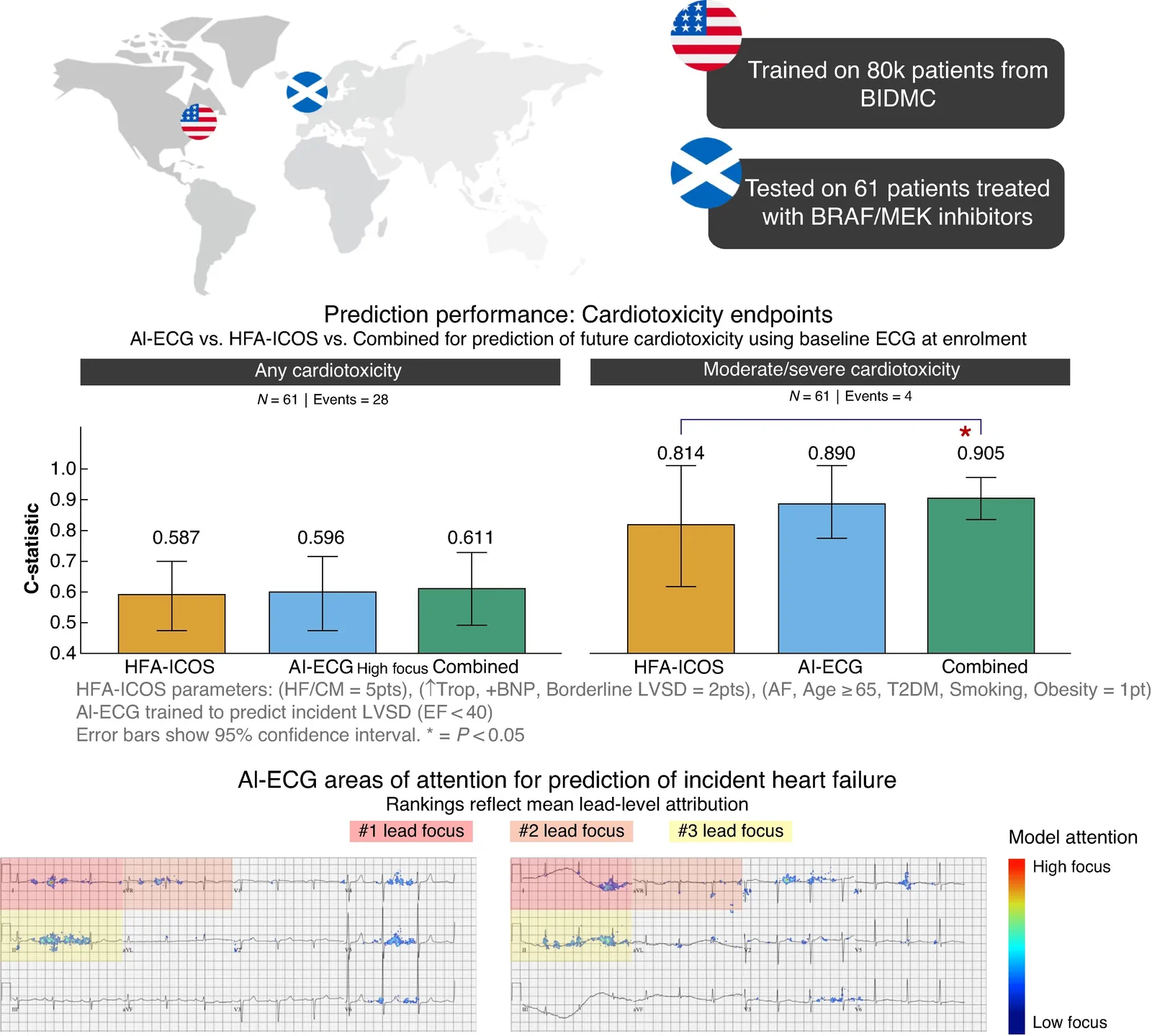
AI-ECG performance predicting BRAF/MEK inhibitor-associated cardiotoxicity. The AI-ECG model trained in a secondary care US cohort was tested on a Scottish cohort of melanoma patients receiving cancer therapy. Bars display C-statistics (error bars: 95% CI) comparing HFA-ICOS (orange), AI-ECG (blue), and the combination of HFA-ICOS and AI-ECG (green). Gradient-based saliency maps (bottom) show model attention overlaid on two baseline ECGs; the top-3 leads by mean attribution across all ECGs were Lead I, aVR, and Lead II. AI-ECG, artificial intelligence-enhanced electrocardiography; Heart Failure Association-International Cardio-Oncology Society; CI, confidence interval.

We also evaluated an incident hypertension model from the AIRE platform, a common side effect in this cohort (46%). Neither the combination of clinical variables nor the AI-ECG model showed strong predictive capacity for this endpoint (C-statistics ∼0.50–0.60). This lack of ECG signal may reflect a pathophysiology driven by acute vascular toxicity rather than pre-existing myocardial vulnerability.

Our findings have important implications for cardio-oncology practice. While the HFA-ICOS score remains a robust tool for baseline stratification, its predictive ceiling is limited by the variables currently available in the electronic health record. The addition of AI-ECG—a low-cost, universally available biomarker—significantly refined risk prediction for the most severe cardiac events. Specifically, the AI model successfully identified the small subset of patients destined for overt LVEF decline, a group that warrants intensive surveillance (e.g. echocardiography at 4 weeks) as suggested by our prior work.^10^ Conversely, the lack of signal for mild toxicity suggests that Grade 1 events may be stochastic or distinct from the pathophysiology leading to overt failure.

Limitations include the small sample size and low event rates, necessitating cautious interpretation; these also precluded time-varying analyses and rendered reclassification analyses unreliable. The AIRE model was trained in a US secondary care population, and while external validation in a distinct Scottish cancer cohort suggests generalisability, replication in larger, multicentre cardio-oncology cohorts with longer follow-up and serial AI-ECG analyses is warranted.

In conclusion, AI-ECG demonstrates a trend towards additive prognostic value beyond HFA-ICOS for predicting moderate/severe CTRCD in patients receiving BRAF/MEKis. Integration into baseline assessment could enable more targeted surveillance of patients at the highest cardiotoxicity risk.

## Data Availability

The Scottish data supporting this study cannot be made publicly available due to patient consent and ethical restrictions but may be made available by the corresponding author upon reasonable request and subject to institutional and ethical approvals. The Beth Israel Deaconess Medical Center dataset is restricted because of ethical limitations.
